# Actinorhodopsin: an efficient and robust light-driven proton pump for bionanotechnological applications

**DOI:** 10.1038/s41598-025-88055-8

**Published:** 2025-02-03

**Authors:** Nooraldeen Ayoub, Nadia Djabeur, Daniel Harder, Jean-Marc Jeckelmann, Zöhre Ucurum, Stephan Hirschi, Dimitrios Fotiadis

**Affiliations:** 1 Institute of Biochemistry and Molecular Medicine, University of Bern, CH-3012 Bern, Switzerland; 2 Present Address: Department of Biochemistry, University of Oxford, Oxford, OX1 3QU UK

**Keywords:** Membrane proteins, Cryoelectron microscopy

## Abstract

Actinorhodopsins are encoded by a distinct group of microbial rhodopsin (MR) genes predominant in non-marine actinobacteria. Despite their role in the global energy cycle and potential for bionanotechnological applications, our understanding of actinorhodopsin proteins is limited. Here, we characterized the actinorhodopsin RlActR from the freshwater actinobacterium *Rhodoluna lacicola*, which conserves amino acid residues critical for light-driven proton pumping found in MRs. RlActR was efficiently overexpressed in *Escherichia coli* in milligram amounts and isolated with high purity and homogeneity. The purified RlActR absorbed green light and its primary proton acceptor exhibited a mildly acidic apparent p*K*_*a*_. Size-exclusion chromatography of RlActR purified in the relatively mild and harsh detergents 5-cyclohexyl-1-pentyl-β-D-maltoside and n-octyl-β-D-glucopyranoside revealed highly homogeneous oligomers and no disruption into monomers, indicating significant robustness of the RlActR oligomer. Cryo-electron microscopy and 2D classification of protein particles provided a projection structure identifying the oligomeric state of RlActR as a pentamer. Efficient establishment of a proton gradient across lipid membranes upon light illumination was demonstrated using RlActR-overexpressing *E. coli* cells and reconstituted RlActR proteoliposomes. In summary, these features make RlActR an attractive energizing building block for the bottom-up assembly of molecular systems for bionanotechnological applications.

## Introduction

Rhodopsins are an extensive and diverse superfamily of light-sensitive membrane proteins found throughout all domains of life^1,2^ as well as in giant viruses^3^. Tens of thousands of entries for rhodopsins can be found in the National Center for Biotechnology Information (NCBI; USA) gene database, the majority of which correspond to microbial rhodopsins (MRs). MRs can be found in various natural niches from the tropics^4^ to the poles^5,6^, saline^7,8^ to freshwaters^9,10^, and soils^11,12^ to plant leaf surfaces^13^. They also prevail in various harsh environments such as hypersaline^14,15^ and hot waters^16,17^. This apparent diversity and ubiquitous nature^18^ of MRs attests to a physiological importance of these proteins in sunlit environments^19^. Structurally, rhodopsins are characterized by a seven transmembrane α-helix (TMH) topology (helices often designated A to G)^1^. MRs typically have their N-termini located extracellularly and C-termini intracellularly, whereas heliorhodopsins display an inverted topology, with N-termini located intracellularly and C-termini extracellularly^20^. The conserved seven-TMH scaffold is customized for various protein functions and corresponding physiological roles^21^. These roles involve photoenergy transduction by light-driven ion pumping (e.g., of H^+^, Cl^–^ and Na^+^ ions) to establish cell-energizing electrochemical gradients, and photoreceptive signaling to regulate cellular responses to photic cues (light-gated ion channels, transducer-coupled sensory rhodopsins and enzyme rhodopsins)^20–22^. Rhodopsin photosensitivity arises when the apoprotein (opsin) covalently binds the co-factor retinal in a binding pocket via a Schiff base linkage. This bond occurs with the side chain of a conserved lysine residue located on the seventh TMH (i.e., helix G) and yields the photoactive retinylidene holoprotein, rhodopsin^20^. In MRs, photon capture by the bound retinal chromophore triggers the rapid and efficient photoisomerization of retinal from all-*trans* to 13-*cis* configuration, initiating ion pumping or signaling^1^. Thermal re-isomerization of retinal occurs in the final steps of the photocycle without its release from the opsin, in contrast to the process in mammalian rhodopsins^1,23^. Co-factor binding and its interactions with the opsin uniquely tune the wavelength at which visible light absorption is maximal (λ_max_)^1^. Color tuning in MRs is important for adaptation of the host organism to the light conditions of its specific natural living environment^24^, optimizing the efficiency of the light-harvesting mechanism^25^. On the other hand, in synthetic biology, stable, efficient and spectrally tunable MRs are highly sought after as modular building blocks for functionalizing artificial, light-driven molecular systems engineered for various bionanotechnological applications^26,27^.

The first discovered MR is bacteriorhodopsin (BR)^15^, a prototypical light-driven proton pump from the haloarchaeon *Halobacterium salinarum*. Several decades of BR research have shaped it into a valuable and pioneering model rhodopsin^28^. In the year 2000, the green-light absorbing proteorhodopsin (GPR) from the genome of uncultured marine γ-proteobacteria was discovered as the first bacterial rhodopsin^29^. Today, GPR represents the archetype for bacterial light-driven proton pumps^30,31^. More surveys and field expeditions quickly provided evidence for thousands of other proteorhodopsin (PR) variants widely distributed across seas and oceans^32–34^, but also in non-marine^34,35^ and terrestrial^36^ ecosystems. PR-based phototrophy is highlighted as a simple, yet influential global phenomenon next to the complex chlorophyll-based photosynthetic systems once thought to be the only solar energy-harvesting mechanism in marine environments^18,19^.

In a bioinformatic endeavor by Sharma et al.^37^ three phylogenetic clades (called LG1, LG2 and PCL1) distinct from the common PR-like clade were discovered, mainly from non-marine metagenomic sequences. The identified genes were eventually named ‘actinorhodopsins’ (ActRs) based on phylogenomic analyses of genes adjacent to these novel sequences, revealing strong affiliation with *Actinobacteria*^37^. *Actinobacteria* is one of the largest phyla in the domain Bacteria and is traditionally associated with terrestrial and freshwater ecosystems, and increasingly with marine environments^38,39^. Indeed, independent reports support the thriving of ActRs-harboring actinobacteria with decrease in salinity^40,41^. More ActR genes were later found in preidentified and cultured freshwater actinobacteria, and in environmental DNA samples retrieved from other freshwater sites^42^. Primary sequence analyses of selected ActRs revealed conservation of amino acid residues crucial for proton pumping in MRs, and no association with photosensory transducer protein genes, suggesting a putative proton pump functionality^37,42^.

Despite being widespread in actinobacteria, only a few ActR proteins so far have undergone biochemical, biophysical and functional characterization. *Rhodoluna lacicola* MWH-Ta8 is an actinobacterium that was isolated from Lake Taihu, China^43^ and found to harbor an ActR gene^42^. Initial characterization of *R. lacicola* ActR (RlActR) involved its visualization in the membrane of *E. coli* by total internal reflection fluorescence (TIRF) microscopy after heterologous expression^44^. Functional assays confirmed the predicted proton pumping activity of RlActR in live *E. coli* and *R. lacicola*^45^. Soon after, an ActR encoded in the closely related freshwater actinobacterium *Rhodoluna planktonica* (RpActR) was shown to have similar functional properties, e.g., retinal binding, visible light absorption and proton pumping activity^46^. Structural insights into ActRs also remain scarce apart from the oligomeric state of QsActR (*Quadrisphaera* sp. R2A-380-A) and KrActR (*Kineococcus radiotolerans*) determined by high-speed atomic force microscopy to be pentamers^47^. However, QsActR and KrActR seem to phylogenetically cluster within a clade different from that of RlActR and RpActR. Specifically, the former two can be found closer to NQ-type rhodopsins (Na^+^ and Cl^–^ pumps)^48^, while the latter two are members of the broader xanthorhodopsins (XR) family^48^ hypothesized to have a secondary pocket that binds an antenna carotenoid^49^ such as salinixanthin or echinenone, occupying XR^50^ and *Gloeobacter* rhodopsin (GR)^51^, respectively.

In this study, we focus on the heterologous overexpression, and biochemical, biophysical, functional and structural characterizations of the actinorhodopsin from *R. lacicola*. We report high-yield protein production in *E. coli*, affinity purification for pure and homogeneous protein as well as successful functional reconstitution of RlActR into liposomes. The latter demonstrated efficient establishment of a proton gradient across lipid membranes upon light illumination by this light-driven proton pump. Additionally, we provide insights into the oligomeric state and projection structure of RlActR using cryo-electron microscopy (cryo-EM). In summary, our results showcase RlActR as an attractive light-driven proton pump for bionanotechnological applications^26,27^.

## Results and discussion

### Comparison of RlActR amino acid sequence with other MRs

**Fig. 1:**
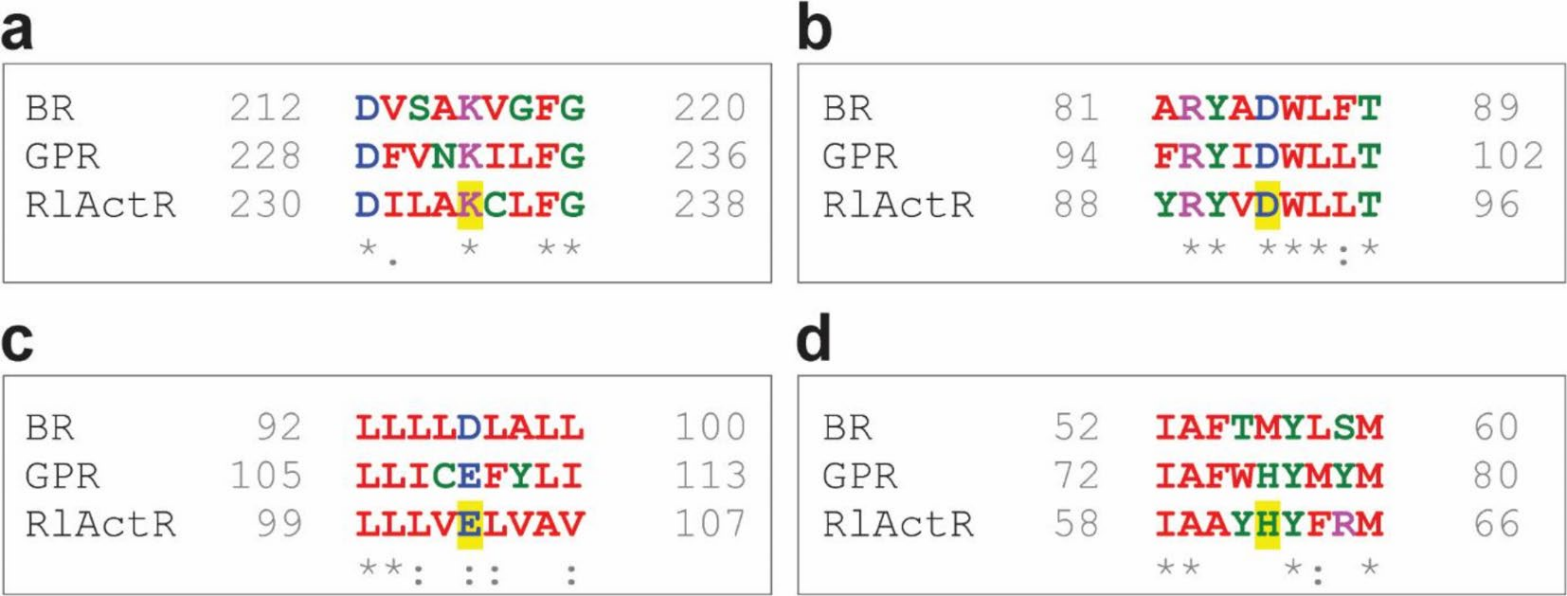
Selected key functional regions from the multiple amino acid sequence alignment of BR, GPR and RlActR (Fig. S1). Residues compared for conservation are the characteristic Schiff base-forming lysine (**a**), the primary proton acceptor (**b**), the primary proton donor (**c**), and a proton acceptor-modulating histidine (**d**). The representatives of these four residues in the RlActR amino acid sequence are shown with a yellow background. Physicochemical properties of all amino acids in the alignment are color-coded with red (hydrophobic), blue (acidic), magenta (basic) and green (hydroxyl, thiol or amide group containing side chains, and histidine and glycine). Consensus symbols are shown under the aligned residues and are an asterisk (fully conserved residue), a colon (strongly similar properties) and a period (weakly similar properties).

Pairwise amino acid (AA) sequence alignment of RlActR against the light-driven proton pumps BR or GPR, prototypical representatives from two different phylogenetic clades other than that of RlActR, shows that both share an AA sequence identity of ∼28% with RlActR. However, a difference is found in the AA sequence similarities, which is higher for GPR (∼47%) than for BR (∼39%), reflecting more similar physicochemical properties of the AAs between RlActR and GPR. This observation aligns with the fact that RlActR and GPR are bacterial proteins, whereas BR is an archaeal protein. Notably, pairwise AA sequence alignment of RlActR with same-family members XR and GR shows an average identity of approximately 42% and similarity of around 59%. A multiple AA sequence alignment of RlActR, BR and GPR (Fig. S1) revealed conservation of key AA residues important for proton pumping. These include (i) K234: the central lysine residue likely forming the retinal Schiff base (Fig. 1a), (ii) D92: the putative primary proton acceptor to which the proton of the Schiff base is transferred after retinal photoisomerization (Fig. 1b), (iii) E103: the putative primary proton donor enabling re-protonation of the Schiff base^1,31,52^ (Fig. 1c) and (iv) H62: a histidine residue characteristic to PR and known to elevate the p*K*_a_ of the primary proton acceptor through hydrogen-bond interaction in PR^30,53^ (Fig. 1d).

### Expression, purification, and biochemical and biophysical characterization of RlActR

**Fig. 2:**
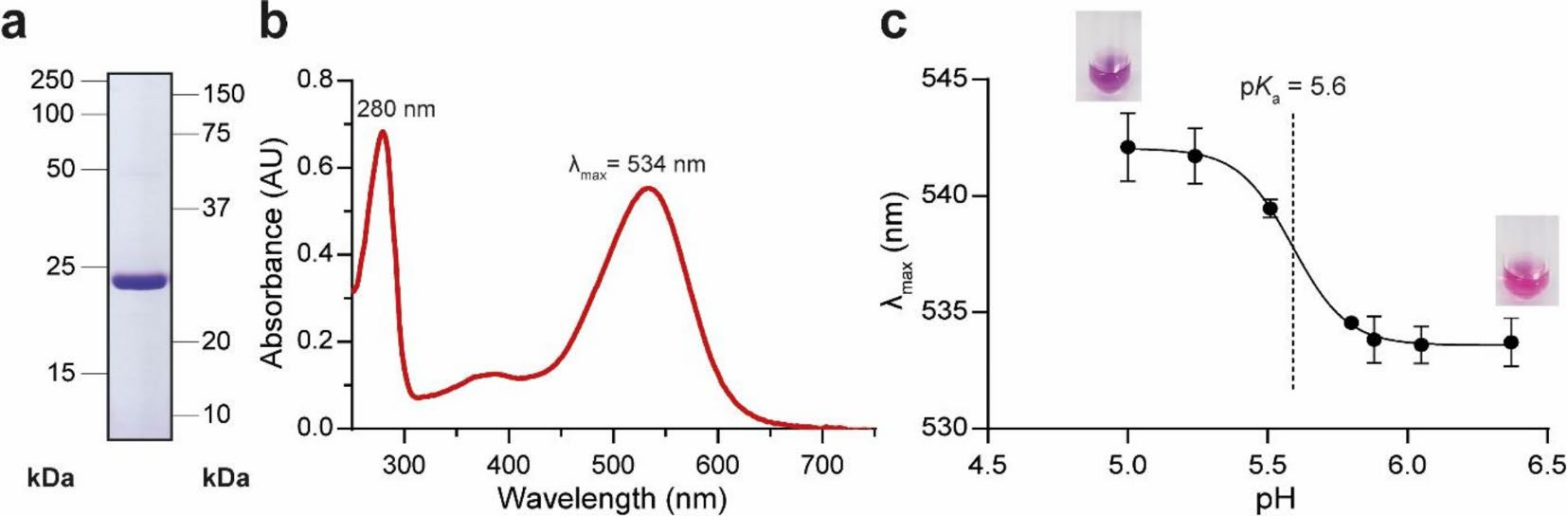
SDS-PAGE analysis and absorption spectrum of Cymal-5-purified RlActR, and spectrophotometric p*K*_a_ determination of the primary proton acceptor. (**a**) 13.5% SDS/polyacrylamide gel loaded with 6 µg of purified 5×His-tagged RlActR. The full (uncropped) gel is shown in (Fig. S2a). (**b**) Representative UV/Vis absorption spectrum of RlActR. The retinal Schiff base displays maximal absorption of visible light at 534 nm. (**c**) Spectrophotometric pH titration of purified RlActR. The plot combines results from three independent titration experiments with protein from independent purifications. For every experiment, each datapoint is an average of triplicate samples measured at specified pH levels. Error bars are standard error of the mean.

The codon-optimized RlActR gene encoding a C-terminal 5×His-tagged was cloned and heterologously overexpressed in *E. coli*. Ni-NTA purification of RlActR yielded highly pure protein when solubilized and purified in the nonionic detergent 5-cyclohexyl-1-pentyl-β-D-maltoside (Cymal-5; Fig. 2a). Despite a theoretical molecular weight of ∼30 kDa for the monomeric RlActR, the purified protein migrated faster in the SDS-PAGE gel, i.e., around 24 kDa, an anomalous migration also observed with various other membrane proteins^54–57^. Using the described expression and purification procedure (Materials and Methods), RlActR could be purified in Cymal-5 at a yield of 8 ± 0.04 mg per liter of bacterial cell culture (*n* = 3). The recovered yield is high compared to that of its counterpart, GPR, purifiable at ∼1.5 mg per liter of culture^54^, reflecting the significantly higher amount of RlActR overexpressed in the bacterial membranes during cell culture.

Retinal-bound MRs have characteristic optical absorption spectra and λ_max_^1^. UV/Vis absorption spectra acquired for purified RlActR revealed a characteristic retinal Schiff base absorption maximum in the visible range with λ_max_ = 534 nm (Fig. 2b). This confirmed RlActR as a green-light-absorbing MR like its bacterial and archaeal homologs GPR (525 nm)^31^ and BR (568 nm)^58^. The λ_max_ value we obtained for RlActR is the same as that of RpActR, which shares ∼81% identity and ∼89% similarity in AA sequence with RlActR^46^.

The interaction of the primary proton acceptor with the proximally located, photon-absorbing retinal Schiff base can be used experimentally to determine its apparent p*K*_a_. This is due to the spectral shifts that occur in response to pH change and thus the (de)protonation of the primary proton acceptor^59,60^. pH titration of purified RlActR between pH 6.4 and 5.0 resulted in a visible color transition from pink to purple with a ∼8 nm spectral shift in λ_max_ from 534 to 542 nm as pH becomes progressively more acidic (Fig. 2c). Redshifting of λ_max_ in response to acidification is reported for light-driven proton pumps and ranges from less than 10 nm in RlActR to ∼20 nm in GPR^61,62^ and ∼40 nm in BR^63^. Interestingly, compared to its closely related homolog, RpActR, which exhibits a significant redshift of ~ 34 nm^46^, RlActR shows a less sensitive and narrower spectral response. The apparent p*K*_a_ value of D92, the putative primary proton acceptor of RlActR, is represented in the half-maximal of the sigmoidal curve obtained from the spectral pH titration of RlActR and was calculated to be 5.6 (Fig. 2c). Despite their contrasting spectral responses to protonation, the apparent p*K*_a_ values found for RlActR and RpActR are similar with 5.8 for RpActR^46^. Finally, compared to the model archaeal and bacterial light-driven proton pumps, the p*K*_a_ of RlActR, which is more similar to GPR (∼7.5)^53^, lies between that of BR (∼2.6)^64^ and GPR.

**Fig. 3:**
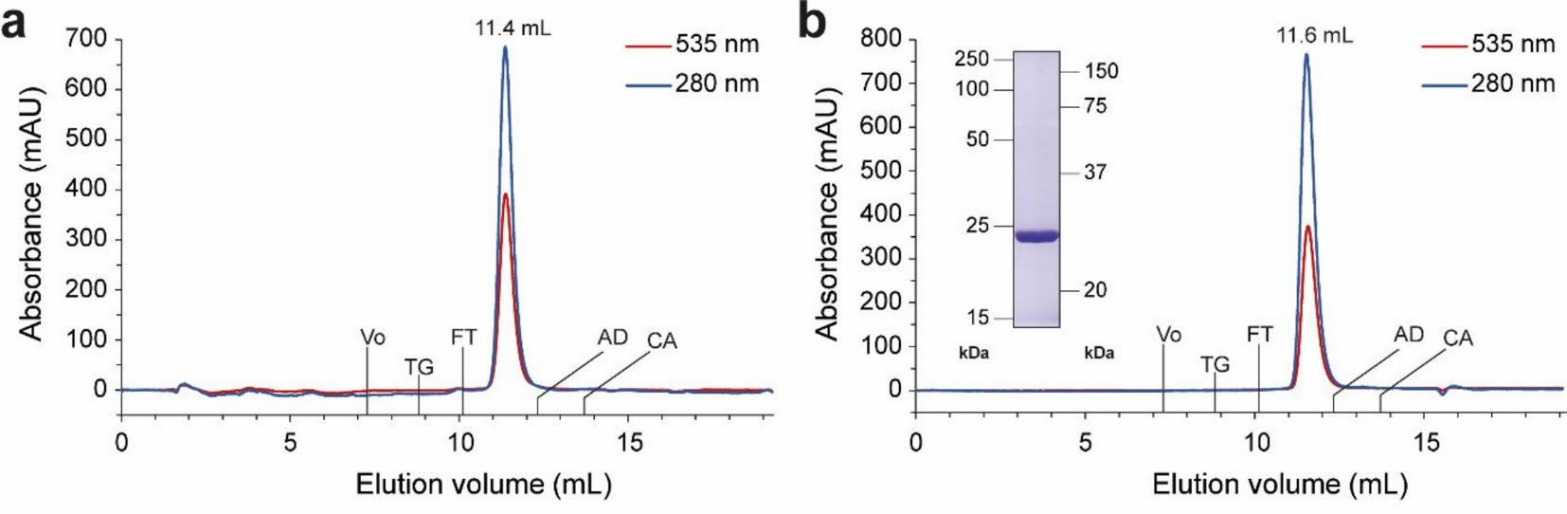
SEC analysis of RlActR purified in Cymal-5 (**a**) or OG (**b**). Absorbance during SEC was measured at 280 and 535 nm. A Superdex 200 Increase 10/300 GL column (GE Healthcare) was used. The elution volumes of RlActR in the different detergents are indicated as well as those of the void volume (V_o_; determined with blue dextran, 2000 kDa) and molecular weight marker proteins, i.e., thyroglobulin (TG, 669 kDa), ferritin (FT, 440 kDa), aldolase (AD, 158 kDa) and conalbumin (CA, 75 kDa). The inset in (**b**) displays an SDS-PAGE gel of RlActR purified in OG. The protein band (6 µg loaded) migrates at ~ 24 kDa on the 13.5% SDS/polyacrylamide gel. See Fig. S2b for full (uncropped) gel.

We performed size-exclusion chromatography (SEC) to assess the homogeneity of the purified RlActR protein and estimate its oligomeric state. RlActR purified in Cymal-5 appeared as a highly homogeneous population, as indicated by the single, sharp absorption peak at an elution volume (V_e_) of 11.4 mL (Fig. 3a). We have previously observed a similar SEC elution peak for Cymal-5-purified GPR (V_e_ = 11.5 mL) using the same SEC column type^61^. Column calibration with known molecular weight (MW) markers allowed the calculation of an apparent MW of 226 kDa for the RlActR protein-lipid-detergent complex, a value comparable to that reported previously for the pentameric GPR protein-lipid-detergent complex (204 kDa)^61^. The comparable MWs of the RlActR and GPR monomers, calculated from their amino acid sequences (i.e., MW_RlActR_ ∼30 kDa and MW_GPR_ ∼26 kDa), suggest a pentameric assembly for RlActR in Cymal-5. However, unlike RlActR, Cymal-5-purified GPR exhibits heterogeneity, with a minor population existing in a lower oligomeric form (V_e_ = 14 mL), previously ascribed to monomeric GPR^61^. Furthermore, when purified in the relatively harsh detergent n-octyl-β-D-glucopyranoside (OG) and analyzed again on the same SEC column, the GPR pentamer dissociated into a homogeneous population eluting at V_e_ = 13.9 mL (MW_app_ = 73 kDa) (Fig. S3), indicative of dissociation into the monomeric GPR form. Conversely, SEC analysis of RlActR purified in OG (Fig. 3b; purification yield 10 ± 0.6 mg from 1 L of bacterial culture (*n* = 3)) revealed an elution peak similar to that for RlActR purified in Cymal-5 (Fig. 3a) and with a calculated apparent MW of 206 kDa (Fig. 3b). Moreover, the sample retains its highly homogeneous state as seen from the sustained peak breadth and symmetry. A similar SEC elution volume and homogeneity of RlActR when purified in OG compared to Cymal-5 demonstrates the remarkable stability of the RlActR oligomer even in a harsh detergent and highlights a rigid nature. OG, known for its relatively high critical micellar concentration, is recognized as an effective membrane protein extraction agent, with the potential to disrupt oligomeric states and affect the stability of various proteins^65^. Indeed, previous works have reported OG-induced monomerization of both trimeric BR^66^ and pentameric GPR^67^.

### Photoactivity of RlActR

**Fig. 4:**
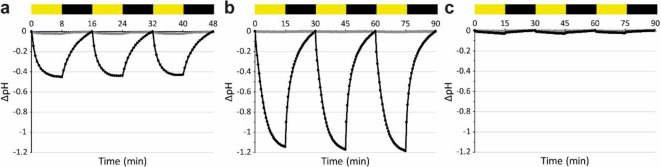
Photoactivity experiments with RlActR-expressing *E. coli* cells (**a**) and RlActR reconstituted in DOPC liposomes without (**b**) and with (**c**) 50 µM CCCP in 2% (v/v) DMSO. The alternating yellow and black rectangles represent intervals of illumination and darkness (each either 8 min for *E. coli* cells or 15 min for proteoliposomes). Each light-dark cycle (*E. coli*, 16 min; proteoliposomes, 30 min) yields a negative peak reflecting outward proton pumping during the light interval and regression of proton flow during the dark interval. The pH at 0 min was ∼6.1 for cells and ∼7.1 for proteoliposomes. The gray trace is the background signal for the unbuffered measuring solutions.

Light-driven proton pumping activity of RlActR was assessed using a standardized assay of light-induced pH change^68^ with suspensions of live bacteria expressing RlActR or purified RlActR protein reconstituted into 1,2-dioleoyl-*sn*-glycero-3-phosphocholine (DOPC) proteoliposomes. *E. coli* cells expressing RlActR prompted reproducible pH decreases and increases in response to exposure to intervals of white light and darkness, respectively (Fig. 4a). One cycle of light-dark exposure (one peak) yielded a maximum pH change (ΔpH) of about − 0.45 units. The negative value of ΔpH indicates a net proton translocation from the inside to the outside of the cell. These results demonstrate that RlActR is a functional light-driven proton pump like BR and GPR. The extracellular acidification signal observed with RlActR-expressing *E. coli* cells (Fig. 4a) was higher than with GPR-overexpressing bacterial suspensions (ΔpH about − 0.27^68^). As described above, a higher protein expression yield per liter of cell culture was obtained for RlActR compared to GPR. Therefore, for the same number of live *E. coli* cells expressing RlActR or GPR, a superior RlActR photoactivity signal might be expected due to the higher number of RlActR copies pumping across the *E. coli* plasma membrane. However, a quantitative comparison of proton transport across lipid membranes upon light illumination by light-driven proton-pumping MRs expressed in *E. coli* is challenging due to the complexity of the living system. This complexity includes variations in the protein expression levels of the respective MRs being compared, bacterial adaptation during measurements over time and other influencing factors. Therefore, RlActR and GPR proteoliposomes were used to compare their light-driven proton-pumping functions.

Illumination of RlActR proteoliposomes resulted in relatively strong extravesicular acidification (Fig. 4b) and indicates an outward proton pumping direction. The signal was reproducible over multiple light-dark cycles. To further support RlActR as a proton pump that builds electrochemical proton gradients across lipid membranes, we added the proton ionophore carbonyl cyanide 3-chlorophenylhydrazone (CCCP) to the RlActR proteoliposomes. Upon addition of CCCP, the proton gradient across the lipid membrane dissipated, reducing the pH signal to near background levels (Fig. 4c). As CCCP was dissolved in dimethyl sulfoxide (DMSO; 2% (v/v) final concentration), we also measured the activity of RlActR proteoliposomes with only DMSO. No significant difference in light-driven pumping activity of RlActR was detected in the presence or absence of DMSO (Fig. S4a). Next, we compared the functionality of RlActR to GPR proteoliposomes prepared using the same reconstitution approach and investigated by the same photoactivity assay setup (see Materials and Methods). Unlike reconstituted GPR, which achieved a ΔpH of −0.7 (Fig. S4b), RlActR proteoliposomes demonstrated a more pronounced ΔpH of −1.2 (Fig. 4b), indicating the superior ability of reconstituted RlActR to establish a proton gradient across a lipid membrane upon light illumination. We then investigated the orientation distribution of reconstituted RlActR and GPR in proteoliposomes. To this aim, we specifically targeted by carboxypeptidase Y (CPY)^69^ the hydrolysis of RlActR and GPR C-termini potentially located in the extravesicular medium and thus accessible for the enzymatic cleavage. Gel electrophoretic analysis of CPY-treated RlActR and GPR proteoliposomes revealed no change in the protein migration pattern compared to untreated samples (Fig. S5a,b), indicating a directed reconstitution with virtually all C-termini of RlActR and GPR located inside the vesicular lumen and thus inaccessible for digestion. As a positive control for the cleavage of extravesicular C-termini of reconstituted MRs^69^, CPY-treated BR proteoliposomes showed a change in the gel migration pattern as a cleaved subpopulation is resolved (Fig. S5c). Altogether, our data demonstrate that the actinobacterial light-driven proton pump RlActR can be successfully reconstituted into DOPC liposomes, where it efficiently establishes a significant proton gradient across the lipid membrane upon light illumination. Furthermore, our findings show that RlActR is uniformely oriented in the vesicular membrane in a right-side-out orientation, ensuring optimal proton pumping efficiency. This is also observed in reconstituted GPR (Fig. S5b) and with *E. coli* expressing RlActR and GPR (see above).

### Projection structure and oligomeric state of RlActR

**Fig. 5:**
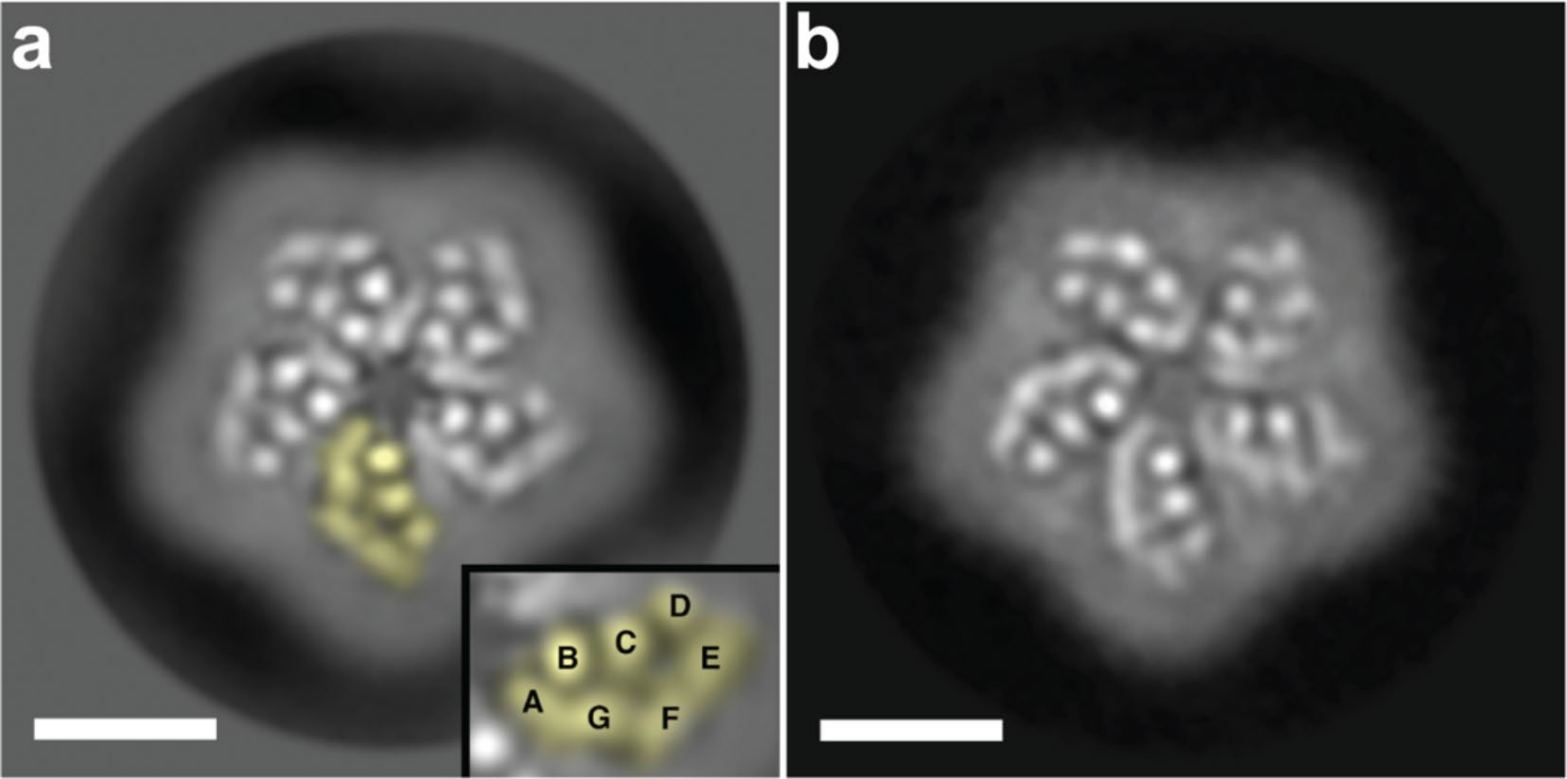
Projection structures of purified RlActR (**a**) and GPR (**b**) determined by single particle cryo-EM. The pentameric nature of the RlActR oligomer is clearly discerned. One monomer is colored in yellow and displayed magnified as an inset. Transmembrane α-helices of RlActR are tentatively labeled (A–G). The projection maps of RlActR and GPR are at 4 and 5.7 Å resolution and were calculated from 53,376 and 1355 particles, respectively. The scale bars represent 4 nm.

SEC analysis of purified RlActR protein indicated that it exhibits a single oligomer form (Fig. 3). To determine the oligomeric state of RlActR, cryo-EM of purified protein was performed. Single particle analysis from electron micrographs provided a 4 Å projection structure (Fig. 5a) and clearly unveiled that RlActR forms pentamers. This finding is in line with the oligomeric states of distantly related QsActR and KrActR^48^ previously determined by high-speed atomic force microscopy^47^. The projection structure provided well-defined or continuous densities (Fig. 5a) and closely resembles those of BR^70^ and sensory rhodopsin II^71^, previously determined by electron crystallography of 2D protein crystals. Using these projection structures as a reference, we tentatively assigned the three well-resolved densities in each monomer to the three perpendicular TMHs B, C and D, while the continuous densities were attributed to the four tilted TMHs A, E, F and G (Fig. 5a; inset). Furthermore, this observed projection structure and TMH arrangement in the RlActR monomer is similar to the projection structure of GPR from cryo-EM single particle 2D class averages^30^ (Fig. 5b).

## Conclusion

In this work, we characterized the actinorhodopsin protein RlActR from the freshwater actinobacterium *Rhodoluna lacicola*. It conserves hallmark residues critical for the light-driven proton-pumping functionality of MRs. We report high heterologous overexpression yields and protein purity using immobilized metal-ion affinity purification. Similar to the model light-driven proton pumps BR and GPR, isolated RlActR contains bound chromogenic cofactor retinal and absorbs visible green light. The primary proton acceptor of purified RlActR was found to have a mildly acidic apparent p*K*_a_ of 5.6. Size-exclusion chromatography analysis of purified RlActR revealed an oligomeric population of high homogeneity. Oligomeric RlActR exhibits remarkable stability as it resists the relatively harsh detergent OG, known to monomerize BR and GPR. Single-particle cryo-EM analysis of the stable RlActR oligomer yielded a 4 Å projection structure revealing a pentameric assembly. Finally, the efficient establishment of a proton gradient across lipid membranes was demonstrated upon light illumination using RlActR-overexpressing *E. coli* cells and successfully reconstituted RlActR proteoliposomes. Our findings establish RlActR as a promising candidate for bottom-up assembly of synthetic molecular systems, serving as a robust and powerful energizing building block.

## Materials and methods

### Amino acid sequence analysis and alignments of selected MRs

The amino acid sequence of RlActR (UniProt ID: C0K2L3) was aligned against BR (UniProt ID: P02945), GPR (UniProt ID: Q6J4G7), XR (UniProt ID: Q2S2F8) or GR (UniProt ID: Q7NP59) using the pairwise sequence alignment tool EMBOSS Needle (version 6.6.0; https://www.ebi.ac.uk/Tools/psa/emboss_needle/)^72^ to compare percent identities and similarities. RlActR, BR and GPR amino acid sequences were aligned using the multiple sequence alignment tool Clustal Omega (version 1.2.4; https://www.ebi.ac.uk/Tools/msa/clustalo/)^73^ to investigate conservation of key rhodopsin amino acid residues in distantly related MRs. Jobs were submitted with default settings. BR N-terminal propeptide was truncated to match common residue numbering in the literature.

### Cloning and overexpression of RlActR and GPR

The actinorhodopsin gene from *Rhodoluna lacicola* strain MWH-Ta8 (RlActR; UniProt ID: C0K2L3) was codon-optimized for expression in *E. coli* and synthesized (GenScript; Table S1). It was cloned using 5’-HindIII and 3’-XhoI restriction sites into the pZUDF21-5×His vector^54^. The resulting construct encodes for recombinant RlActR and the C-terminal amino acid sequence Leu-Glu-Gly followed by the 5×His-tag. Competent cells of *E. coli* C43(DE3) strain were transformed with the pZUDF21-RlActR-5×His construct by the heat shock method. A highly RlActR-expressing clone was identified by colony selection combined with Western blotting. Cells of the selected clone were grown overnight in a suspension of Luria-Bertani (LB) medium supplemented with 100 µg/mL ampicillin in an orbital shaker at 37 °C and 180 rpm (Multitron, Infors HT). The overnight culture was used for inoculation and cultivation of several 2 L cultures. At OD_600_ of 0.3–0.4, the temperature was reduced to 18 °C and overexpression of RlActR was induced at OD_600_ of 0.75 with 250 µM isopropyl-β-D-1-thiogalactopyranoside (IPTG) and 5 µM of the cofactor *all-trans* retinal (dissolved in ethanol). The cultures were further grown overnight at 18 °C. For GPR, cloning and overexpression were done as described previously^74^.

### Isolation of *E. coli* membranes

RlActR-overexpressing *E. coli* cells were harvested by centrifugation for 6 min at 10,000 × g and 4 °C (HiCen XL centrifuge, Herolab). Cell pellets from 10 L culture were resuspended in a total of 450 mL of membrane wash buffer (50 mM Tris-HCl pH 8 adjusted at 4 °C, 450 mM NaCl). The cell suspension was centrifuged again for 8 min at 10,000 × g and 4 °C, and the total pellet was finally resuspended in 400 mL of the same buffer by shaking for at least 1 h at 200 rpm and 4 °C (Aqua-Shaker, Haska AG, Bern). The final cell suspension was frozen at -20 °C overnight. The frozen *E. coli* cells were thawed in a water bath at 30 °C and incubated with ∼50 mg of chicken egg white lysozyme powder (Apollo Scientific Ltd) and shaking for 10 min at 200 rpm and 4 °C (Aqua-Shaker, Haska AG, Bern). The cells were further lysed by five passages in a Microfluidizer M-110P (Microfluidics) at 1,500 bar. The lysate was centrifuged for 6 min at 10,000 × g and 4 °C to remove cellular debris and unbroken cells. Membranes in the supernatant containing recombinant RlActR were washed by ultracentrifugation for 1.5 h at 150,000 × g and 4 °C, followed by resuspension and homogenization with membrane wash buffer. The membranes were washed two more times by ultracentrifugation for 1 h at 150,000 × g and 4 °C. The final pellet was resuspended and homogenized in purification buffer (20 mM HEPES-NaOH pH 7.5 adjusted at room temperature (RT), 300 mM NaCl, 10% (v/v) glycerol). Membranes were stored at -80 °C until further use as 3 mL aliquots corresponding to membranes from 1 L of cell culture. *E. coli* membranes containing GPR were isolated as described previously^74^, excluding Tris-(2-carboxyethyl)-phosphine.

### Immobilized metal-ion affinity chromatography (IMAC) purification of RlActR and GPR

A 3 mL aliquot of membranes containing recombinant RlActR was thawed at RT and solubilized overnight in the dark on a turnover shaker at 4 °C in 7 mL of purification buffer containing 3% (w/v) Cymal-5 (Anatrace) or OG (Glycon Biochemicals). Next morning, the sample was ultracentrifuged for 40 min at 100,000 × g and 4 °C. The supernatant was added to 6.5 mL purification buffer (containing 60 mM imidazole) and 0.5 mL bed volume of ProteinIso Ni-NTA Resin (TransGen) pre-equilibrated with the same buffer. RlActR was bound to the resin at RT for 2 h on a turnover shaker in the dark and then transferred to a gravity-flow column (Promega). The resin was washed three times with 7 mL purification buffer containing 60 mM imidazole and either 0.25% (w/v) Cymal-5 or 1% (w/v) OG. RlActR was eluted as 200 µL fractions (unless stated otherwise) with 1.5-2 mL of elution buffer A (20 mM HEPES-NaOH pH 7.5 adjusted at RT, 150 mM NaCl, 10% (v/v) glycerol, 200 mM L-histidine, 0.25% (w/v) Cymal-5 or 1% (w/v) OG) added in 0.5 mL increments. The protein concentration was determined spectrophotometrically using a NanoDrop OneC instrument (Thermo Scientific), the absorbance of RlActR at 280 nm, and the theoretical mass extinction coefficient of RlActR at 280 nm (1.376 mL⋅mg ^− 1^⋅cm ^− 1^). For cryo-EM, RlActR was purified with a minor modification (see ‘Cryo-EM sample and grid preparation’ section below). For GPR, solubilization and purification with the detergent OG was done as described previously^74^.

For SDS-PAGE, IMAC-purified RlActR samples were mixed with 5x sample loading buffer (100 mM Tris-HCl pH 6.8, 25% (v/v) glycerol, 5% (w/v) SDS, 0.1% (w/v) bromophenol blue) for a final dye concentration of 2x. Wells of a 13.5% SDS/polyacrylamide gel were loaded with 6 µg of protein. The gel was stained by standard Coomassie Brilliant Blue R-250 staining.

### UV/Vis spectra of RlActR

Purified RlActR (in Cymal-5) was run through a 2 mL Zeba Spin Desalting Column (7 kDa molecular weight cut-off; Thermo Scientific) pre-equilibrated with exchange buffer A (20 mM HEPES-NaOH pH 7.5 adjusted at RT, 150 mM NaCl, 10% (v/v) glycerol, 0.25% (w/v) Cymal-5) to remove L-histidine. 0.5 mg/mL protein samples were prepared in UV-Vis cuvette (UVette, Eppendorf) and were used to acquire UV/Vis spectra (range: 250–750 nm) using a NanoDrop OneC instrument (Thermo Scientific).

### Spectrophotometric pH titration of RlActR

A series of UV/Vis spectra scans and λ_max_ determinations were performed at different pHs using Cymal-5-purified RlActR. Four RlActR IMAC elutions (100 µL fractions) with the highest concentrations (darkest red color) were combined and run through a 2 mL Zeba Spin Desalting column (7 kDa molecular weight cut-off, Thermo Scientific) pre-equilibrated with exchange buffer B (5 mM HEPES-NaOH pH 7.5 adjusted at RT, 150 mM NaCl, 0.25% (w/v) Cymal-5) to diminish the buffering capacity of HEPES and remove L-histidine. The sample was then ultracentrifuged for 10 min at 200,000 × g and 4 °C, and the supernatant was diluted to 1 mg/mL protein concentration using exchange buffer B. From this, aliquots were each mixed with a corresponding assay buffer solution (125 mM acetate buffer, 150 mM NaCl, 0.25% (w/v) Cymal-5) to obtain a series of 300 µL assay samples in 100 mM acetate buffer at 0.2 mg/mL protein concentration, and with a final pH of 6.4, 6.1, 5.9, 5.8, 5.5, 5.2 or 5.0 (adjusted at RT). UV/Vis spectra were recorded for all assay samples using a NanoDrop OneC instrument (Thermo Scientific) with a sampling interval of 0.5 nm in polystyrene cuvettes (Sarstedt, Germany). λ_max_ was determined for each sample and plotted against pH of that sample using GraphPad Prism 9 software. Finally, the datapoints were analyzed with the GraphPad Prism function “Sigmoidal dose-response (variable slope)”. From the nonlinear-regression curve fit, the half-maximal was computed, which represents an apparent p*K*_a_ value of the primary proton acceptor of RlActR.

### Analytical SEC

Prior to SEC, IMAC elution fractions of Cymal-5- or OG-purified RlActR, or of OG-purified GPR were combined and ultracentrifuged for 10 min at 200,000 × g and 4 °C. 220 µL at ∼0.75–1.5 mg/mL of the sample of interest (i.e., RlActR or GPR) was prepared by diluting a supernatant aliquot with elution buffer A. The diluted sample was injected through a 200 µL loop into an Äkta purifier (GE Healthcare) fitted with a Superdex 200 Increase 10/300 GL column pre-equilibrated with SEC buffer (20 mM Bis-Tris propane (BTP)-HCl pH 7.5 adjusted at 4 °C, 150 mM NaCl, 0.25% (w/v) Cymal-5 or 1% (w/v) OG). The UV/Vis detector of the system was set to 280 nm and either 535 nm for RlActR or 525 nm for GPR.

### Photoactivity assay of RlActR in *E. coli*

RlActR-overexpressing *E. coli* C43(DE3) cells aliquoted from an overnight culture (see overexpression protocol above) were centrifuged for 10 min at 5000 × g at RT and gently resuspended in 0.8 mL unbuffered measuring solution A (150 mM NaCl pH 7.4 adjusted at RT) to an OD_600_ of 40. The cells were washed a second time by centrifugation and resuspension. Proton-pumping photoactivity of RlActR overexpressed in living bacterial cells was measured in a clear 2 mL tube using the setup described previously^68^. Briefly, the 2 mL tube containing the cells was placed in a water bath (18 °C) and gently stirred magnetically. Extracellular pH was measured over successive light and dark intervals using a micro pH-electrode (InLab Micro Pro, Mettler Toledo) with an integrated temperature sensor. The sample was illuminated using a 2 W, 3000 K, warm white LED lamp (JANSJÖ, IKEA)^74^ and the duration of the light-dark intervals were controlled using a generic digital time switch. Automated pH recording was performed every 30 s by the software LabX direct pH 2.3 connected to the pH-meter device (Mettler Toledo). pH data were collected over four light-dark cycles (four peaks) with each constituting 8 min of light followed by 8 min of darkness. The cells were allowed an 8 min darkness adaptation period prior to the first light-dark cycle (1st peak). Background signal was measured using 0.8 mL of unbuffered measuring solution A. The experiment was constantly protected from exterior light. For a comparable assay and set-up to measure proton and ion transport activity of microbial rhodopsins expressed in *Escherichia coli* cells, see following reference^75^.

### Reconstitution of RlActR, GPR and BR into liposomes

400 µL of 25 mg/mL 1,2-dioleoyl-*sn*-glycero-3-phosphocholine (DOPC; Avanti Polar Lipids) dissolved in chloroform was dried in a COREX glass test tube using a nitrogen gas stream. Lipid drying and chloroform removal was completed by placing the tube in a vacuumed desiccator overnight at RT (in the dark). Next day, the lipid was hydrated by adding 2 mL of lipid hydration buffer (20 mM HEPES-KOH pH 7.5 adjusted at RT, 100 mM KCl) and mixing for 10–15 min at 700 rpm and RT on a Thermomixer (Eppendorf). For RlActR and GPR, the formed liposomes were destabilized by adding 162 µL of a 10% (w/v) OG solution for a final OG concentration of ∼0.75% (w/v), and further shaking for 10–15 min at 700 rpm and RT. IMAC elution fractions of OG-purified RlActR or GPR were buffer exchanged using a 2 mL Zeba Spin Desalting Column (7 kDa molecular weight cut-off; Thermo Scientific) pre-equilibrated with exchange buffer C (20 mM HEPES-KOH pH 7.5 adjusted at RT, 100 mM KCl, 5% (v/v) glycerol, 1% (w/v) OG). The sample was then centrifuged for 10 min at 20,000 × g and 4 °C. 580 µL of 0.75 mg/mL RlActR or GPR, prepared by diluting the supernatant using exchange buffer C, was added into the suspension of dispersed and destabilized liposomes (i.e., added protein amount = 435 µg; lipid: protein ratio = 23; final OG concentration = 0.8% (w/v)). The suspension was extruded 19 times through a Whatman polycarbonate membrane (pore size: 0.2 μm) using a Mini-Extruder (Avanti Polar Lipids). The extruded sample was loaded into a 14 kDa molecular weight cut-off cellulose dialysis tubing (Membra-Cel, Carl Roth) and dialyzed overnight with slow magnetic stirring against 2 L of detergent-free dialysis buffer (20 mM HEPES-KOH pH 7.5 adjusted at RT, 100 mM KCl). Next morning, the dialyzed sample was split into four tubes for efficient washing by ultracentrifugation for 20 min at 200,000 × g and 4 °C. Each pellet was resuspended in 800 µL of unbuffered measuring solution B (100 mM KCl pH 7.5 adjusted at RT). Four washes were performed and after the last spin, the pellets were combined and resuspended in 150 µL of unbuffered measuring solution B. Purple membranes from *H. salinarum* S9 strain containing BR were prepared as described previously^69,76^ and solubilized at 1 mg/mL protein concentration in lipid hydration buffer containing 3% (w/v) OG (final solubilization mix = 400 µL) for 3 h at 700 rpm and RT (Thermomixer, Eppendorf). Solubilized BR was separated by ultracentrifugation for 1 h at 100,000 × g and 4 °C. The supernatant (∼200 µg BR) was added to 10 mg of priorly hydrated, extruded and destabilized DOPC lipid. To destabilize, 83.3 µL of 10% (w/v) OG was added (final OG concentration = 0.4% (w/v)). The mix was then shaken for 30 min at RT. The following overnight dialysis and washing were done as described above for the RlActR and GPR proteoliposomes.

### Photoactivity assay of RlActR and GPR proteoliposomes

The ability to establish a pH difference across lipid membranes upon light illumination within a given time span was assessed using RlActR and GPR proteoliposomes in a setup similar to that used for cells. The 150 µL proteoliposomes suspension was loaded into a 600 µL transparent glass tube and maintained in a cooling water bath (Julabo F10, Gemini) at 18 °C by gentle magnetic stirring. The pH of the extravesicular medium was measured over successive light and dark intervals by a micro pH-electrode (InLab Ultra Micro-ISM, Mettler Toledo) combined with a temperature sensor (ATC Probe, Mettler Toledo) inserted into the cooling vessel. Light-dark intervals were generated using a spectrally tunable light engine (Spectra Tune Lab, LEDMOTIVE Technologies) configurable by the PC software µWave STLAB 1.3.0. During a light interval, the sample is illuminated with a beam in the range of 500–600 nm (i.e., green light) and at 50% output flux (∼467 lm; optical power ∼1038 mW). The pH was recorded automatically every 30 s by the software LabX direct pH 2.3 (Mettler Toledo) connected to the pH-meter device (SevenCompact, Mettler Toledo). Data was recorded over four light-dark cycles (four peaks) with each constituting 15 min of an illumination interval followed by a 15 min darkness interval. Prior to the first light-dark cycle (1st peak), an initial 15 min period of sample adaptation to darkness was allowed. For background signal measurement, 150 µL of unbuffered measuring solution B was used. The whole setup was constantly protected from exterior light. Next, photoactivity of the proteoliposomes was measured in the presence of only 2% (v/v) DMSO as a control for the proton gradient dissipation across the membrane experiment with CCCP. DMSO was washed away (see washing procedure above) and photoactivity was then remeasured in the presence of 50 µM CCCP (dissolved in 2% (v/v) DMSO) added from a 2.5 mM stock (in 100% (v/v) DMSO).

### Photoactivity data correction

Raw pH data were corrected as described previously^68,77^. Briefly, a continuous piecewise linear function, the slope of which is defined for each peak by two sequential starting points of illumination, is calculated and subtracted from the raw data to attain pH drift correction. The first recorded light-dark cycle (1st peak) of the corrected data was then discarded due to adaptation period of the system during the first illumination interval and the last three peaks are presented in the figures.

### Carboxypeptidase Y (CPY) digestion assay

16 µg of RlActR, GPR or BR from the prepared proteoliposomes was taken and completed to 20 µL with unbuffered measuring solution B. Then, 20 µL of 2 mg/mL CPY (Sigma) in 100 mM MES-KOH pH 6.75 adjusted at RT, or CPY-free MES buffer was added. The samples were incubated overnight at 25 °C. Finally, 1–2 µg of the corresponding MR was mixed with 5x sample loading buffer (final dye: 3.75x), heated for 5 min, at 700 rpm and 80 °C (Thermomixer, Eppendorf), and loaded on a precast 12% NuPAGE Bis-Tris gel (Invitrogen) for electrophoresis using MOPS/SDS running buffer (Fig. S5). The full (uncropped) gel of Fig. S5 is shown in Fig. S6.

### Cryo-EM sample and grid preparation

IMAC purification of RlActR was slightly modified for cryo-EM data acquisition by integrating a buffer-exchange during purification. To this end, 7 mL of 20 mM BTP-HCl pH 7.5 adjusted at 4 °C, 150 mM NaCl, 2.5 mM L-histidine, 0.25% (w/v) Cymal-5, was used in the 2nd and 3rd wash during purification. To elute, elution buffer B (20 mM BTP-HCl pH 7.5 adjusted at 4 °C, 150 mM NaCl, 200 mM L-histidine, 0.25% (w/v) Cymal-5) was used. The freshly purified sample was diluted to 2.5 mg/mL and 3 µL were applied onto glow-discharged holey carbon R1.2/1.3 (Quantifoil) copper grids. Glow discharge was performed for 10 s at 10 mA, 200 mV and 0.25 mbar. The vitrification process was performed in a Vitrobot Mark IV apparatus (Thermo Fischer Scientific, The Netherlands). In brief, grids were incubated for 30 s, and excess liquid blotted off for 5 s applying a blotting force of −6 at 4 °C and a relative humidity of about 100% prior to plunging into liquid ethane.

### Cryo-EM data acquisition

Cryo-EM data were collected on a Glacios 2 cryo-transmission electron microscope (Thermo Fischer Scientific, The Netherlands) operated at an acceleration voltage of 200 kV. The nominal defocus used to collect data ranged from ∆z = −1 to −3 μm, using the latest-generation direct electron detector CMOS Falcon 4i camera with Selectris X energy filter (slit width 10 eV), operating in electron event representation (EER) mode^78^. The Falcon 4i was calibrated at a nominal magnification of 121,317× resulting in 1.154 Å pixel size at the specimen level. A total of 1,021 movies were collected with the program SerialEM^79^. The camera was set up to collect 1,809 raw EER frames in counting mode with a total exposure time of 5.7 s, resulting in a total dose equivalent to 50 electrons/Å^2^ per exposure.

### Image processing and single particle analysis

Beam-induced motion correction of dose-fractionated and gain-corrected EER movies were performed using MotionCor2 (version 1.4)^80^. Initial estimation for the contrast transfer function (CTF) was carried out with Gctf (version 1.6)^81^. Images displaying strong drift, astigmatism greater than 500 Å and maximum CTF resolution worse than 5 Å were excluded from further processing. Subsequently, a total of 1,073,447 particles were auto-picked using CrYOLO (version 1.5)^82^ with the pre-trained general model and extracted from the 987 dose-weighted micrographs with Relion 4^83^. Two-dimensional (2D) particle classifications for projection structure and oligomerization determination were carried out in Relion 4. After 2D classification, we selected a 2D class representing the top view, projection structure of the oligomer, which comprised a total of 53,376 particles (Fig. 5a). For the GPR projection structure (Fig. 5b), data were from our previous work^30^: see there for sample preparation, cryo-EM data acquisition, image processing and single particle analysis.

## Electronic supplementary material

Below is the link to the electronic supplementary material.

Supplementary Material 1

## Data Availability

The datasets generated and analyzed during the present study are available from the corresponding author on reasonable request.
