# Supplementary material for: Actinorhodopsin: an efficient and robust light-driven proton pump for bionanotechnological applications
Source: Sci Rep. 2025 Feb 3;15:4054. doi: 10.1038/s41598-025-88055-8 (PMC11790970; doi:10.1038/s41598-025-88055-8)
Supplement: Supplementary file 1 — Supplementary Material 1 [file 41598_2025_88055_MOESM1_ESM.pdf]

Supplementary Information

Actinorhodopsin: An Efficient and Robust Light-driven Proton Pump For Bionanotechnological Applications

Nooraldeen Ayoub, Nadia Djabeur, Daniel Harder, Jean-Marc Jeckelmann, Zöhre Ucurum, Stephan Hirschi[1] & Dimitrios Fotiadis*

Institute of Biochemistry and Molecular Medicine, University of Bern, CH-3012 Bern, Switzerland

[1] Current address: Department of Biochemistry, University of Oxford, OX1 3QU Oxford, United Kingdom

* To whom correspondence should be addressed:

Dimitrios Fotiadis, Email: dimitrios.fotiadis@unibe.ch

```
BR          -------------------------QAQITGRPEWIWLALGTALMGLGTLYFLVKGMGVS    35
GPR         MGKLLLILGSVIALPTFAAGGGDLDASDYTGV--SFWL--VTAALLASTVFFFVERDRVS    56
RlActR      ----------------MNTLSNALDNGQFNLVYNILSL--GIASMLFTAIFLFVARERVL    42
                                      .: .     : *    * :   :::::*    *

BR          DPDAKKFYAITTLVPAIAFTMYLSMLLGYGLTMVPFGGEQNP---IYWARYADWLFTTPL    92
GPR         -AKWKTSLTVSGLVTGIAFWHYMYMRGVWIE----------TGDSPTVFRYIDWLLTVPL    105
RlActR      -PRYRIAVMVSATVTAIAAYHYFRMFDNFSHAFA--GAENNPDAYNVGYRYVDWLLTVPL    99
                :    ::  * .**   *: *   :                    ** ***:*.**

BR          LLLDLALLVDADQ---GTIL-ALVGADGIMIGTGLVGALTKV---YSYRFVWWAISTAAM    145
GPR         LICEFYLILAAATNVAGSLFKKLLVGSLVMLVFGYMGEAGIMAAWPA-----FIIGCLAW    160
RlActR      LLVELVAVLALAKAAQSSILNRLVPAAAAMIVLGYPGDAKLD-IWGIAPSVWGLLSTIPF    158
            *: ::  ::       .:::  *: .   *:  *  *                 :.

BR          LYILYVLFFGFTSKAES-MRPEVASTFKVLRNVTVVLWSAYPVVWLIGSEGAGIVPLNI-    203
GPR         VYMIYELWAGEGKSACNTASPAVQSAYNTMMYIIIFGWAIYPVGYFTGYLMGDGGS----    216
RlActR      LYILYVLFIELGKSLSR-QSEAVQKKVKILRLLLIATWGVYPITFILAMGTPPGAPFNAS    217
            :*::* *:    ..        * .  : :  : :  *. **: :: .

BR          ----ETLLFMVLDVSAKVGFGLILLRSRAIFGEAEAPEPSAGDGAAATSD    249
GPR         -ALNLNLIYNLADFVNKILFGLIIWNVAVKESSNA---------------    250
RlActR      EFVAREVGYSIADILAKCLFGLIIYSIARIKSAEDDKEFAKAEFKDA---    264
                  : : : *.  *  ****:       .
```

Figure S1: Amino acid sequence alignment of BR (UniProt ID: P02945), GPR (UniProt ID: Q6J4G7) and RlActR (UniProt ID: C0K2L3). BR N-terminal propeptide was truncated to match common residue numbering in the literature. Physicochemical properties of all amino acids in the alignment are color-coded with red (hydrophobic), blue (acidic), magenta (basic) and green (hydroxyl, thiol or amide group containing side chains, and histidine and glycine). Consensus symbols are shown under the aligned residues and are an asterisk (fully conserved residue), a colon (strongly similar properties) and a period (weakly similar properties).

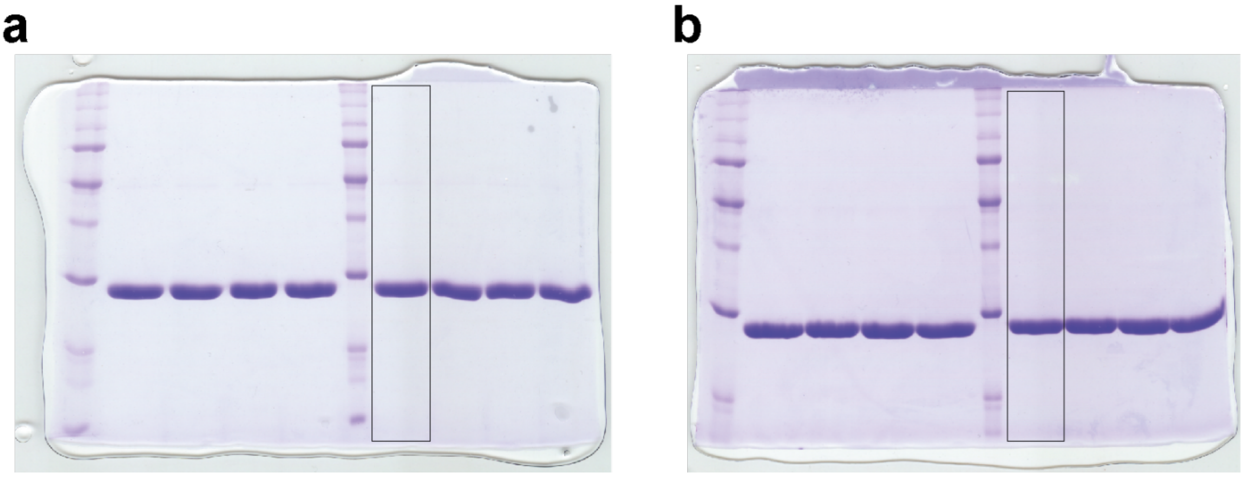

Figure S2: Full (uncropped) SDS-PAGE gels. The lane indicated by the black rectangle in panel **a** is shown in Figure 2a and the lane indicated by the black rectangle in panel **b** is shown in Figure 3b.

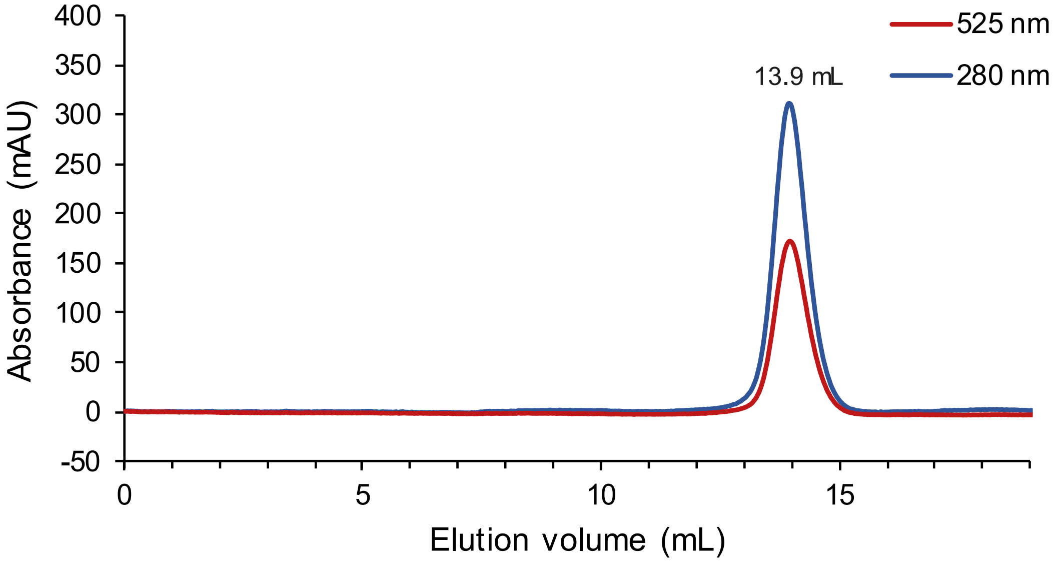

Figure S3: Size-exclusion chromatography (SEC) analysis of green-light absorbing proteorhodopsin (GPR) purified using the detergent OG. Absorbance during SEC was measured at 280 nm and 525 nm. A Superdex 200 Increase 10/300 GL column (GE Healthcare) was used.

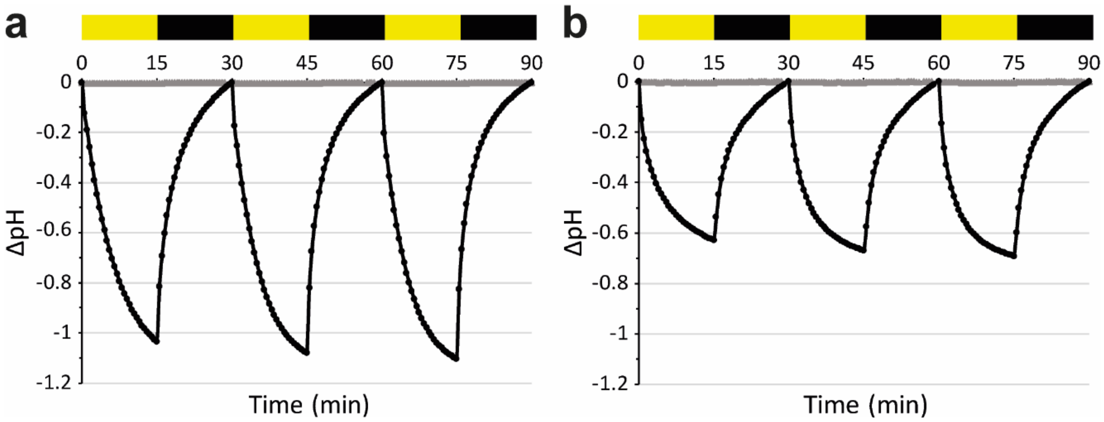

Figure S4. Photoactivity experiments with RlActR reconstituted in DOPC liposomes measured in the presence of 2% (v/v) DMSO (**a**) and with GPR reconstituted in DOPC liposomes (**b**). The alternating yellow and black rectangles represent intervals of illumination and darkness (each is 15 min). Each light-dark cycle (30 min) yields a negative peak reflecting outward proton pumping during the light interval and regression of proton flow during the dark interval. The pH of the samples at 0 min was 7.1−7.3. The gray trace is the background signal recorded for the unbuffered measuring solution.

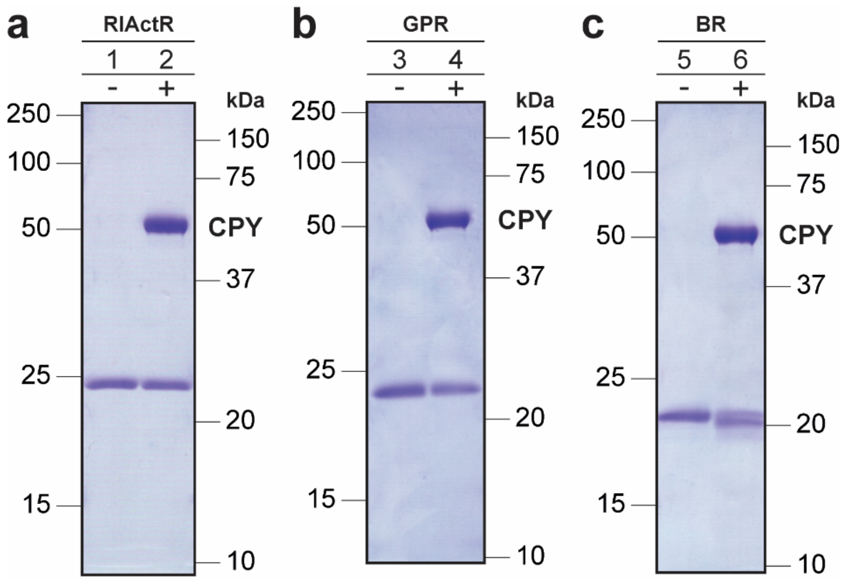

Figure S5. SDS-PAGE analysis of carboxypeptidase Y (CPY)-treated and -untreated proteoliposomes of RlActR (**a**), GPR (**b**) or BR (**c**; positive control). CPY-treated samples (+): lanes 2, 4 and 6. CPY-untreated samples (-): lanes 1, 3 and 5. The CPY protein band is labelled. For each of the microbial rhodopsins, enzymatic digestion experiments were performed for two independent reconstitutions (n = 2). The full (uncropped) gels are shown in Figure S6.

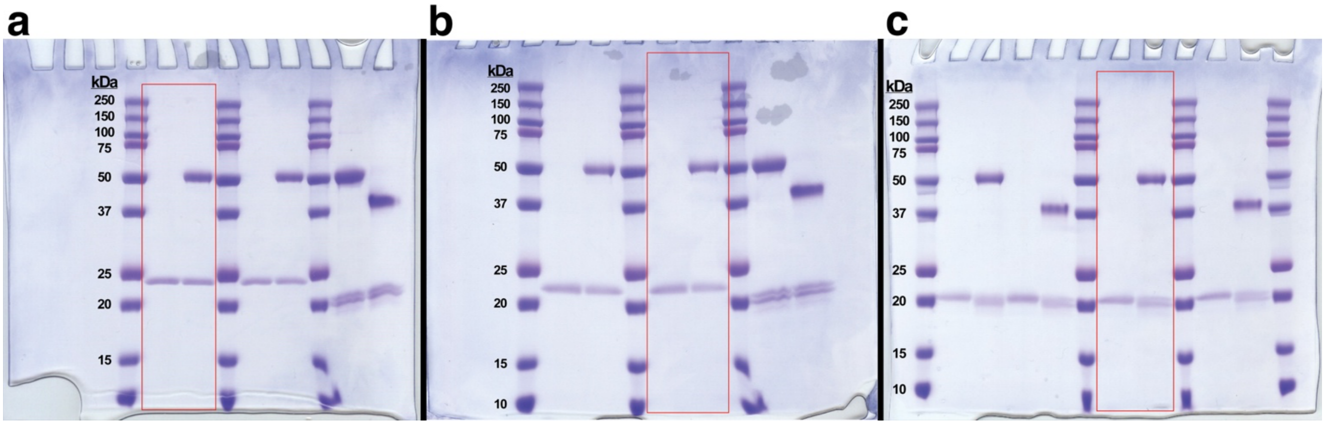

Figure S6: Full (uncropped) SDS-PAGE gels. The lanes marked by red rectangles are displayed in Figure S5, i.e., the marked lanes in panel **a** in Figure S5a, the marked lanes in panel **b** in Figure S5b and the marked lanes in panel **c** in Figure S5c.

Table S1: Synthetic, codon-optimized gene sequence of RlActR for overexpression in *Escherichia coli*.

```
ATGAACACCCTGAGCAACGCGCTGGACAACGGCCAGTTTAACCTGGTGTACAACATCCTGAGCCTGGGCATTGCGAGCATGCT
GTTCACCGCGATCTTCCTGTTTGTGGCGCGTGAGCGTGTTCTGCCGCGTTATCGTATCGCGGTGATGGTTAGCGCGACCGTTA
CCGCGATTGCGGCGTACCACTATTTCCGTATGTTTGATAACTTCAGCCACGCGTTTGCGGGTGCGGAGAACAACCCGGACGCG
TACAACGTGGGCTACCGTTATGTTGATTGGCTGCTGACCGTGCCGCTGCTGCTGGTTGAACTGGTGGCGGTTCTGGCGCTGGC
GAAAGCGGCGCAAAGCAGCATTCTGAACCGTCTGGTGCCGGCTGCGGCGGCGATGATTGTTCTGGGTTATCCGGGCGACGCGA
AGCTGGATATCTGGGGTATTGCGCCGAGCGTGTGGGGCCTGCTGAGCACCATCCCGTTTCTGTACATTCTGTATGTTCTGTTC
ATCGAGCTGGGTAAAAGCCTGAGCCGTCAGAGCGAAGCGGTGCAAAAGAAAGTTAAGATCCTGCGTCTGCTGCTGATTGCGAC
CTGGGGCGTGTACCCGATCACCTTTATTCTGGCGATGGGTACCCCGCCGGGTGCGCCGTTTAACGCGAGCGAGTTCGTGGCGC
GTGAAGTTGGTTACAGCATCGCGGACATTCTGGCGAAATGCCTGTTCGGCCTGATCATTTATAGCATCGCGCGTATTAAGAGC
GCGGAGGACGATAAGGAATTTGCGAAAGCGGAGTTCAAGGATGCG
```
